# The Mechanical Properties of Silicone Rubber Composites with Shear Thickening Fluid Microcapsules

**DOI:** 10.3390/polym15122704

**Published:** 2023-06-16

**Authors:** Chun Wei, Xiaofei Hao, Chaoying Mao, Fachun Zhong, Zhongping Liu

**Affiliations:** Institute of Chemical Materials, China Academy of Engineering Physics (CAEP), Mianyang 621900, China; weichun20@gscaep.ac.cn (C.W.); haoxiaofei163@163.com (X.H.); chaoyingmao@caep.cn (C.M.); zhongfachun@caep.cn (F.Z.)

**Keywords:** shear thickening fluid, silicone rubber, microcapsule, impact resistance behavior

## Abstract

In this study, Sylgard 184 silicone rubber (SylSR) matrix composites with shear thickening fluid (STF) microcapsules (SylSR/STF) were fabricated. Their mechanical behaviors were characterized by dynamic thermo-mechanical analysis (DMA) and quasi-static compression. Their damping properties increased with the addition ofSTF into the SR in DMA tests and the SylSR/STF composites presented decreased stiffness and an obvious positive strain rate effect in the quasi-static compression test. Moreover, the impact resistance behavior of the SylSR/STF composites was tested by the drop hammer impact test. The addition of STF enhanced the impact protective performance of silicone rubber, and the impact resistance increased with the increase of STF content, which should be ascribed to the shear thickening and energy absorption of STF microcapsules in the composites. Meanwhile, in another matrix, hot vulcanized silicone rubber (HTVSR) with a mechanical strength higher than Sylgard 184, the impact resistance capacity of its composite with STF (HTVSR/STF) was also examined by the drop hammer impact test. It is interesting to note that the strength of the SR matrix obviously influenced the enhancement effect of STF on the impact resistance of SR. The stronger the strength of SR, the better the effect of STF on improving the impact protective performance of SR. This study not only provides a new method for packaging STF and improving the impact resistance behavior of SR, but is also beneficial for the design of STF-related protective functional materials and structures.

## 1. Introduction

Shear thickening fluid (STF), as a lightweight, intelligent, and efficient impact protective material, has attracted much research interest [1,2]. It is a solid–liquid suspension system composed ofa high concentration of particles and liquid oligomers. In the steady state, STF appears as a viscous liquid with fluidity, but its viscosity will increase sharply to a solid-like state after being impacted. This liquid–solid conversion is rapid and reversible, accompanied by a large amount of impact energy dissipation. The various mechanisms of the shear thickening phenomenon, such as order-disorder transition theory, hydro-cluster theory, jamming theory, and friction contact theory, have been proposed by researchers [3,4]. Moreover, the reasons for energy dissipation of STFs under impact loading have been considered to be viscous damping [5], fraction between clusters [6], cracking of the jammed network [7,8], and extrusion deformation, cracking, and crushing of particles under high impact pressure [9,10]. Due to their flexible and excellent energy absorption properties, STF-treated high-performance fabrics such as Kevlar and UHMWPE have attracted much attention in applications of body protection and exhibit enhanced bullet-proof and stab-resistant properties [11,12,13].

Since STFs are liquids without a fixed three-dimensional shape under normal conditions and are sensitive to the environment due to their easy moisture absorption, they need to be properly encapsulated in practical applications. To encapsulate STFs, Zhang et al. added a small amount of polyethylene imine into STF and dripped it into an MDI isocyanate prepolymer, and then prepared polyurea-walled macroscopic STF capsules [14]. Zhang Xin et al. further developed three methods to prepare STF microcapsules and realized the reinforcement of the polyurea shells [15]. Liu et al. added a sodium alginate solution and Span 20 to the STF paraffin solution containing Span 80, then the mixture was dropped into anhydrous calcium chloride aqueous solution using a syringe to prepare STF capsules [16]. Kaczorowski et al. adopted polypropylene glycol diacrylate as a monomer to prepare a slightly crosslinked shear thickening liquid organic gel and then embedded it into the uncured polyurethane mixture to form STF/polyurethane elastomer composites [17]. Soutrenon et al. used the vacuum resin infusion method to infiltrate STFs into foam and then covered the outer layer with silicone rubber to achieve the encapsulation of STF/foam composites [18].

Silicone rubber (SR) is a potential material for packaging and storing STFs due to its good barrier performance against water and air and chemical inertness to STFs [19]. Meanwhile, because of its excellent mechanical properties such as high elasticity, low impedance, viscoelasticity, low-temperature resistance, aging resistance, and flexibility, silicone rubber is often used as an impact energy absorption material in the weapon, shipping, electronics, and machinery industries [20]. Developing methods to improve the impact energy absorption performance of silicone rubber materials also has important practical significance in promoting the application of silicone rubber for impact protection.

In our previous work, the impact protective property of the SR matrix composites with shear thickening fluid microcapsules was studied under high-strain-rate loadings. It was found that this composite was a promising flexible material for impact protection due to its flexibility at a low strain rate (10^−3^ s^−1^) but higher stiffness at a high impact loading rate (3500 s^−1^) [21]. In this work, their mechanical properties were characterized by dynamic thermo-mechanical analysis (DMA) measurements, quasi-static compression tests, and drop hammer impact tests. It was found that the addition of STFs can improve the impact protection performance of silicone rubber and the impact resistance increased with the increase of STF content. The composites presented decreased stiffness and an obvious positive strain rate effect in the quasi-static compression test. Notably, the strength of the silicone rubber matrix influenced the enhancement effect of STFs on the impact resistance of silicone rubber.

## 2. Materials and Methods

### 2.1. Materials

Tetraethoxysilane (TEOS, 99.9%) and ammonia water (NH_3_·H_2_O, 25–28%) were purchased from Chengdu Kelong Chemical Reagent Co., LTD. Ethanol (EtOH, 96%) and polyethylene glycol (PEG, Mw = 200) were both purchased from China Sinopharm Chemical Reagent Co., LTD. Sylgard 184 silicone elastomer kit, consisting of a base agent (part A) and a curing agent (part B), is a kind of hydro-silylated liquid silicone rubber purchased from Dow Corning Co., LTD. Methyl vinyl silicone rubber (MVMQ, 110-2, Mw = 6.4 × 10^5^), which contains 0.17 mol% vinyl groups on the backbone chain, was commercially obtained from Dongjue Fine Chemicals (Nanjing). Hydroxyl silicone oil (GY-209-3) was provided by the Chenguang Research Institute of Chemical Industry, China. Dicumyl peroxide (DCP), a vulcanized agentof hot vulcanized silicone rubber, was purchased from Aladdin. The reinforcing filler fumed silica (AS200, hydrophilic) was obtained from Evonik Degussa, Germany.

### 2.2. SiO_2_ Preparation

SiO_2_ was synthesized by a modified Stöber method as follows. Firstly, 16.25 mL ethanol, 9.0 mL ammonia, and 24.75 mL water were introduced into a beaker under magnetic stirring at 800 rpm at room temperature. Then, a mixture of 6 mL TEOS and 44 mL ethanol was quickly added to the breaker. After 5 min, the stirring speed was changed to 400 rpm, and the reaction was maintained for 2 h. Finally, the ethanol in the solution was removed under vacuum and the SiO_2_ was obtained.

### 2.3. STF Preparation

The STF was prepared by dispersing SiO_2_ into a solution of PEG/ethanol under sonication mixing for 4 h, and the amounts were set to be 68:32:600 (weight by weight, *w*/*w*) of SiO_2_/PEG/ethanol. After homogeneous mixing, the ethanol was removed by rotary evaporation, and the viscous STF composed of PEG and SiO_2_ was finally obtained.

### 2.4. SR/STF Composites Preparation

**Figure 1:**
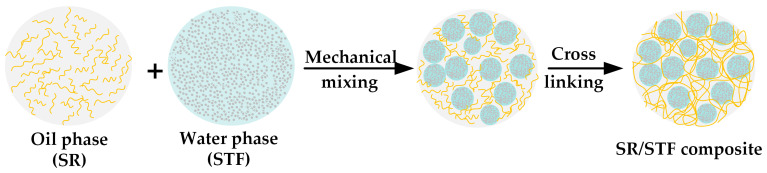
Schematic diagram for preparing the SR/STF composites.

The SR/STF composites were fabricated by emulsifying the STF in silicone rubber and then vulcanizing the mixture. The schematic diagram for preparing the SR/STF composites is depicted in Figure 1.

In detail, when Sylgard 184 silicone rubber was used as the matrix, a certain amount of the STF was added into Sylgard 184 silicone rubber ($m_{A}$:$m_{B}$ = 10:1) and mechanically mixed at 300 rpm for 5 min, and then defoamed by a vacuum oven. After that, the mixture was poured into a mold and cured in an air-dry oven at 80 °C for 2 h, then SylSR/STF composites were obtained. The mass fractions of the STF in the SylSR/STF composites were 0%, 10%, 20%, 30%, and 40%, respectively. For brevity, they were abbreviated as SylSR, SylSR/STF-10, SylSR/STF-20, SylSR/STF-30, and SylSR/STF-40, respectively.

In addition, the hot vulcanized silicone rubber was also adopted as another silicone rubber matrix. The hot vulcanized silicone rubber/STF composite was prepared by homogeneously mixing the hot vulcanized rubber component (methyl vinyl silicone rubber, fumed silica, hydroxyl silicone oil, and DCP with a weight ratio of 100:40:4:2) with STF using a double-roller mixing machine. Then, they were vulcanized in a flat vulcanizing machine at 165 °C for 12 min and the hot vulcanized silicone rubber matrix composites with shear thickening fluid microcapsules were prepared, marked as HTVSR/STF. The vulcanized silicone rubber materials without the STF were also prepared by the same method, and it was labeled as HTVSR.

### 2.5. Characterization

The microstructural characteristics of SiO_2_ were analyzed by field-emission scanning electron microscopy (FE-SEM, UItra55, Carl Zeiss Ltd., Oberkochen, Germany) at a 10 KV acceleration voltage and attenuated total reflection-Fourier transform infrared spectroscopy (ATR–FTIR, Nicolet 800, Thermo Fisher Scientific, Waltham, MA, USA) at a resolution of 4 cm^−1^ for a total of 32 scans with a scan wave between 400 and 4000 cm^−1^. The particle-size distribution was analyzed by Image J. The rheological properties of the STF and PEG were tested using a Kinexus Pro rotary rheometer (Malvern, UK). The diameter of the lamina was 40 mm with a cone angle of 1°. The spacing was 0.03 mm, and the temperature was 25 °C. The microstructures of the composites were characterized using a Axio Lab.A1 (Zeiss, Jena, Germany) optical microscope (OM) with a CCD Camera and FE-SEM. The dynamic thermo-mechanical properties were analyzed using a DMA Q800 (TA, New Castle, DE, USA) in compression mode. It was a cylindrical sample with a 13 mm diameter and 3 mm height. In temperature scan tests, the temperature range was −50~150 °C, with a heating rate of 5 °C/min, frequency of 1 Hz, and amplitude of 5 μm. In frequency scan tests, the frequency range was 0.1–200 Hz, with a temperature of 25 °C and an amplitude of 5 μm. The quasi-static uniaxial compression tests were carried out on Instron-5582 electronic universal testing machine at room temperature. The samples were in cylindrical sizes with a diameter of 29 mm and a height of 12.5 mm. The loading rates of the test were set to be 0.5, 5, 50, and 200 mm/min, respectively, which corresponded to the quasi-static engineering strain rates of 0.00067 s^−1^, 0.0067 s^−1^, 0.067 s^−1^, and 0.267 s^−1^, respectively. The maximum engineering strain of the compressed specimen was about 0.6. The drop weight impact tests were used to evaluate the impact resistance behavior of materials and conducted on the drop hammer impact tester conforming to the EN1621-1-2012 standard [22]. The samples were in rectangular blocks with sizes of 150 mm × 100 mm × 4.80 mm (length × width × thickness). The drop hammer weight was 4.977 kg, the fall height was 42.6 cm, and the impact energy was 20 J.

## 3. Results and Discussion

### 3.1. Microstructural Characteristics of SiO_2_

**Figure 2:**
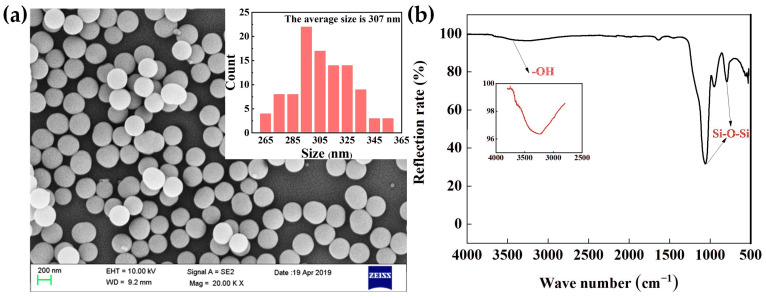
(**a**) SEM micrograph of SiO_2_ particles and their size distribution graph, and (**b**) FTIR curve of SiO_2_.

The microstructure of the SiO_2_ particles and their size distribution was investigated by SEM. As shown in Figure 2a, the SiO_2_ particles were nearly spherical monodisperse with an average size of about 300 nm. The surface state of SiO_2_ particles was characterized via FTIR, and its typical absorbance spectra curve is shown in Figure 2b. The peaks at 794 cm^−1^ and 1058 cm^−1^ were assigned to the symmetric and asymmetric stretching vibrations of Si-O-Si bridges, respectively. The broad peak between 3700 cm^−1^ and 2800 cm^−1^ was the stretching vibration of Si-OH groups [23]. This result indicates that the SiO_2_ prepared in this work is hydrophilic.

### 3.2. Rheological Behavior of STF

**Figure 3:**
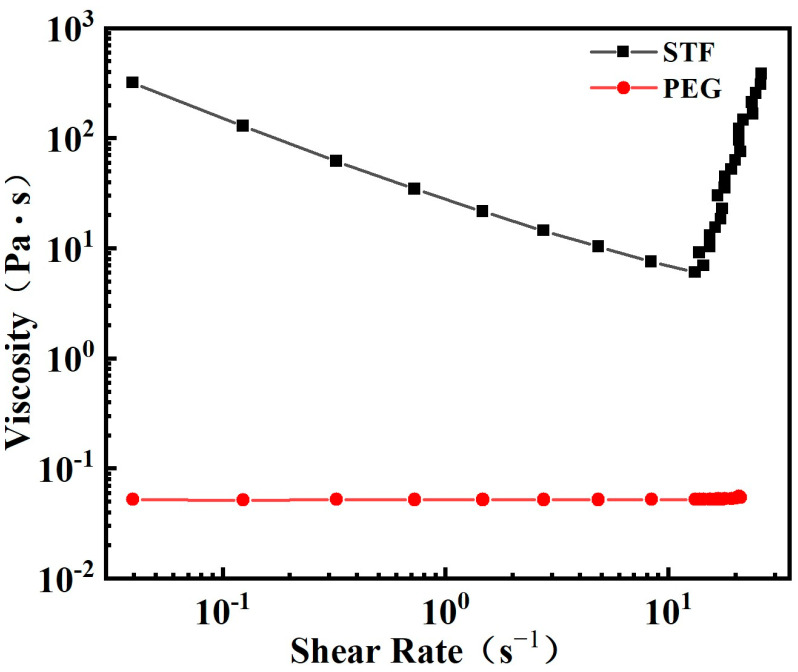
The rheological behaviors of STF and PEG.

Figure 3 presents the rheological behavior of the as-prepared STF and PEG. As a Newtonian fluid, PEG exhibited the same low viscosity value (0.5 Pa·s) at different shear rates. In contrast, the STF showed a shear thinning at low shear rates with the viscosity value decreasing from the initial 318 Pa·s to 6 Pa·s, while when the shear rate increased beyond the “threshold” of 13 s^−1^, the viscosity increased steeply into 381 Pa·s and the STF became extremely viscous. Note that this was not the highest viscosity value for STF; the experiment was stopped automatically at higher strain rates and the viscosity values were not recorded due to the self-protection of the rheometer from high torque forces. This is the typical curve of a discontinuous shear thickening fluid according to previous reports [2,5]. Hence, it should be concluded that the as-prepared STF will undergo a liquid–solid conversion and then dissipate impact energy under the impact loading.

### 3.3. Microstructure of SylSR/STF Composites

**Figure 4:**
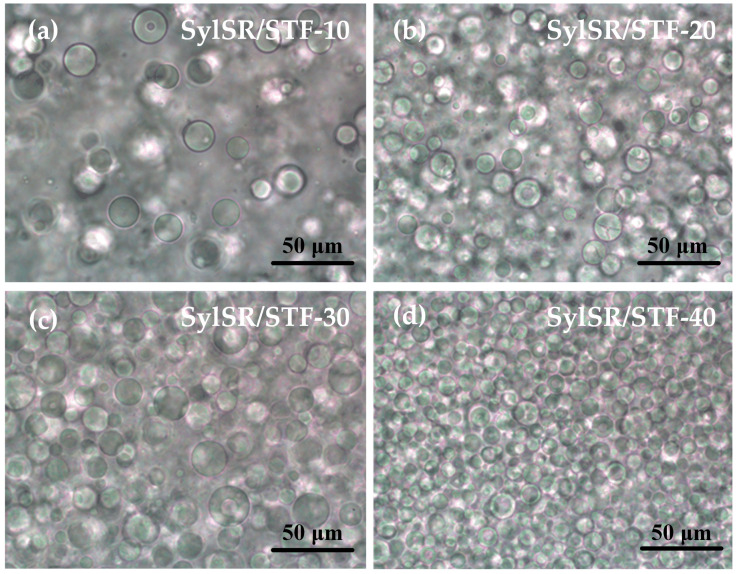
Optical micrographs of SylSR/STF composites with different mass fractions of STF. (**a**) 10%, (**b**) 20%, (**c**) 30%, and (**d**) 40%.

**Figure 5:**
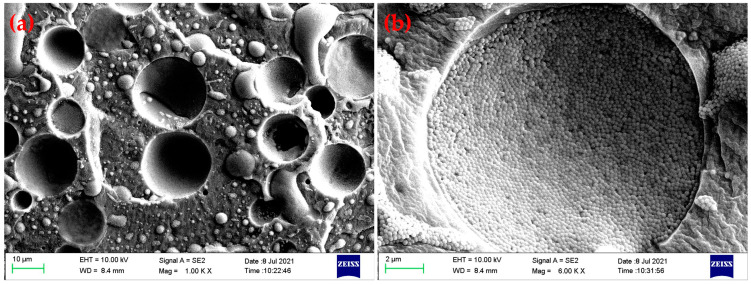
SEM images of (**a**) the fracture surface of a typical SylSR/STF composite and (**b**) the enlarged image of a STF microcapsule.

The microstructure of the SylSR/STF composites was first characterized using an optical microscope. As can be seen from the optical microscopy photos in Figure 4, STF was dispersed in the silicone rubber matrix in the form of spherical microcapsules for all the SylSR/STF composites. The microcapsules in all SylSR/STF composites had a wide diameter distribution from 5 to 25 μm. In particular, the mean diameters of the STF microcapsules for SylSR/STF-10, SylSR/STF-20, SylSR/STF-30, and SylSR/STF-40 were 13.7 ± 5.0, 13.2 ± 4.2, 13.4 ± 4.5, and 10.2 ± 2.1 μm, respectively. To further investigate the STF microcapsules, the SylSR/STF composites were made brittle and broken using liquid nitrogen, and then their fracture surface properties were investigated by FE-SEM. As shown in Figure 5, silica microspheres in the matrix of silicone rubber exhibited a state of aggregation rather than the average distribution state. In particular, it was observed that the silica microspheres were in STF microcapsules (Figure 5b). Therefore, the results indicate the composites prepared in this work were indeed composed of STF microcapsules and silicone rubber matrix, rather than a simple homogeneous mixture of SiO_2_, PEG, and silicone rubber. Since SiO_2_ microspheres and PEG, the components of the STF, are hydrophilic, the STF itself is a hydrophilic liquid. Considering the hydrophobicity of silicone rubber, a water-in-oil emulsion would be formed when the STF and silicone rubber are mixed under mechanical stirring [24]. Then, the water-in-oil configuration could be fixed with the help of the silicone rubber curing process, which resulted in the microstructure formation of STF microcapsules in the silicone rubber matrix.

### 3.4. Mechanical Properties and Impact Resistance Behavior of SylSR/STF Composites

**Figure 6:**
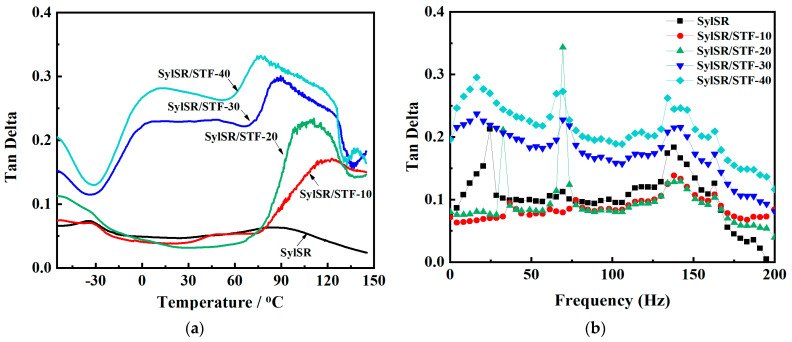
(**a**) Tan δ versus temperature curves and (**b**) Tan δ versus frequency curves of SylSR/STF composites.

Dynamic thermo-mechanical analysis (DMA) measurements were used to investigate the damping properties by calculating the damping factor Tan δ of the SylSR/STF composites at different temperatures and frequencies, and the results are shown in Figure 6. It was found that the values of Tan δ for SylSR/STF-30 and SylSR/STF-40 were higher than that of silicone rubber in the temperature range of −50~150 °C. In addition, at the tested frequency range, 0.1–200 Hz, when the weight fractions of the STF were 10% and 20%, the values of Tan δ of the SylSR/STF composites were slightly lower than silicone rubber. When the mass fractions of the STF were increased to 30% and 40%, the Tan δ values of the SylSR/STF composites were greatly increased. Based on these results, it was concluded that STFs could markedly improve the damping properties of silicone rubber, which means that SylSR/STF composites have a better protection ability than silicone rubber itself when they are applied in changeable temperature environments and complex impact frequency domains.

**Figure 7:**
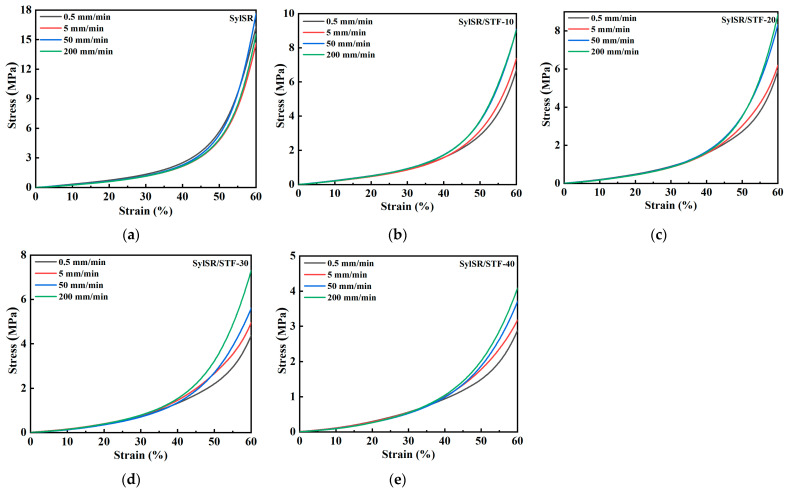
Quasi-static compressive stress–strain curves of all specimens at various strain rates. The specimens were (**a**) SylSR, (**b**) SylSR/STF-10, (**c**) SylSR/STF-20, (**d**) SylSR/STF-30, and (**e**) SylSR/STF-40.

**Figure 8:**
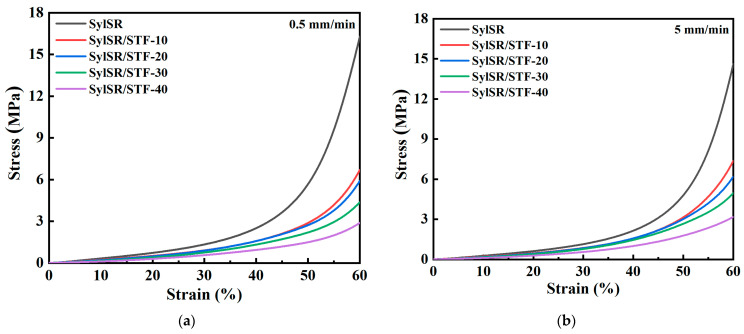

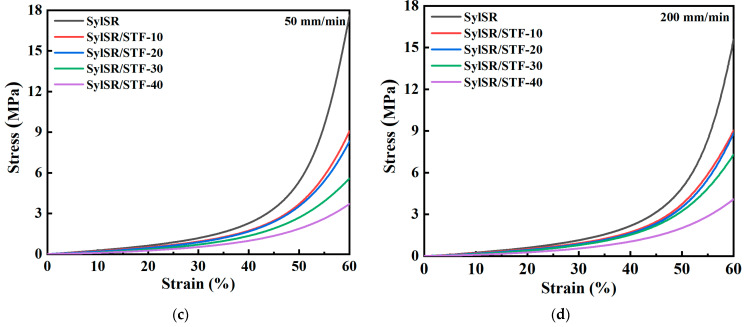
Quasi-static compressive stress–strain curves of all specimens at various strain rates. The loading rates were (**a**) 0.5 mm/min, (**b**) 5 mm/min, (**c**) 50 mm/min, and (**d**) 200 mm/min.

The mechanical behavior of the SylSR/STF composites under low strain rates was studied using compression tests on a universal testing machine. The engineering stress–strain relationships of the SylSR/STF composites under loading rates ranging from 0.5 mm/min to 200 mm/min are shown in Figure 7 and Figure 8. The results indicate that all the five kinds of composites show nonlinear elasticity for strains smaller than 60%. In addition, at the same strain, the stiffness increased with the increase of strain rate, as shown in Figure 7, indicating an obvious positive strain-rate sensitivity of the SylSR/STF composites. As shown in Figure 8, the stiffness of the composite decreased with the increasing mass fraction of the STF microcapsules under low strain rates. According to the mechanical behavior of the STF, below the critical strain rates of the STF it acts as liquid and exhibits slight shear thinning behavior, leading to the weakening effect when its content increases. The lower stiffness of the composite indicates the higher flexibility of the material with the increasing mass fraction of the STF microcapsules, which is strongly required for the design of soft impact protective structures [25].

**Figure 9:**
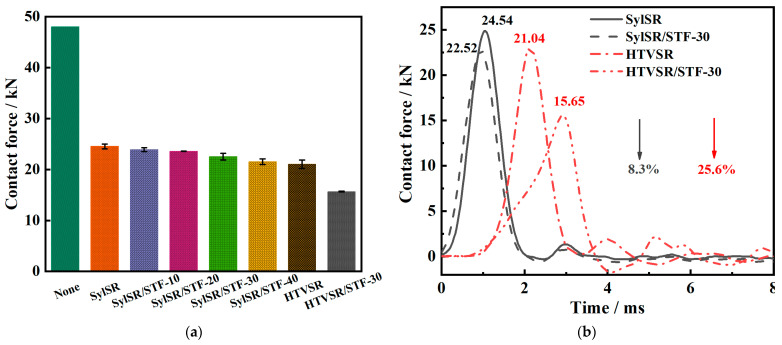
(**a**) Contact force for all specimens tested in drop hammer impact tests, and (**b**) typical contact force–time curves of SylSR, SylSR/STF-30, HTVSR, and HTVSR/STF-30.

The dynamic impact experiments conducted on drop hammer impact tester were used to evaluate the impact resistance behavior of SylSR/STF composites under low-speed impacts. In these experiments, two different kinds of silicone rubber, i.e., hydro-silylated liquid silicone rubber (Sylgard 184) and hot vulcanized silicone rubber (HTVSR), were used as the matrix of the SylSR/STF composites. The experiments were conducted with the same 48 kN initial impact force, and the contact forces tested behind the specimens were adopted to evaluate their impact resistance behaviors. Every specimen was tested at least three times. Figure 9a shows the results of the average value of contact force for the different tested specimens.

For the SylSR/STF composites, with the increase in the STF mass fraction, the contact force was reduced. When the fraction of the STF increased to 30%, the contact force was reduced by 8.3%, from 24.54 kN to 22.52 kN; the typical curves are presented in Figure 9b. This result indicated that the STF can enhance the impact resistance properties of silicone rubber, and the higher the mass fraction of the STF in SylSR/STF composites resulted in a better impact protection effect. Since it was reported that the shear thickening effect of STFs can dissipate impact energy, with an increase in the STF fraction, more energy would be absorbed [5,9,13]. Therefore, SylSR/STF-30 presented a lower value of contact force compared with SylSR itself.

To further evaluate the effect of the mechanical strength of the silicone rubber matrix on the impact resistance of composites with STF microcapsules, another silicone rubber, HTVSR, was selected as a matrix. The mechanical strength of the HTVSR matrix is higher than that of the Sylgard 184 silicone rubber matrix in the SylSR/STF composites [26]. The HTVSR/STF composites presented a significant decrease in the value of the contact force. Their data are also shown in Figure 9a and their typical curves are shown in Figure 9b. The value of the contact force for HTVSR/STF-30 was reduced by 25.6% in comparison with HTVSR, from 21.04 kN to 15.65 kN, and the contact force reduction degree for HTVSR/STF-30 in comparison with HTVSR was almost four times of that of SylSR/STF-30 in comparison with SylSR. Hence, it can be concluded that the strength of the silicone rubber matrix has an obvious influence on the effect of improving the impact protective performance of silicone rubber with the addition ofSTF. The stronger the strength of the silicone rubber, the better the effect of STF on improving the impact-protective performance of the composites. The enhancement effect of STF on the impact resistance of silicone rubber composites should be ascribed to the shear thickening and the energy absorption of STF microcapsulesunder external force impact, which are influenced by the strength of the silicone rubber.

## 4. Conclusions

In this work, SR/STF composites were successfully prepared by a simple mechanical mixing and curing process. STF was dispersed in a silicone rubber matrix in the form of microcapsules, and the mechanical test results showed that the STF improves the impact resistance behavior of silicone rubber. The specific conclusions are as follows:

(1) The damping properties of silicone rubber can be enhanced by the addition of STF and the increase of the fraction of the STF increases the value of Tan δ.
(2) The SR/STF composites presented decreased stiffness and an obvious strain rate effect at low strain rates (from 0.5 mm/min to 200 mm/min) in quasi-static compression experiments.
(3) The addition of STF can improve the impact protection performance of silicone rubber and the impact resistance increased with the increase in the STF mass fraction.
(4) The enhancement effect of STF on the impact resistance of silicone rubber is influenced by the strength of the silicone rubber matrix. The stronger the strength of silicone rubber, the better the effect of STF on improving the impact protective performance of the silicone rubber.

## Data Availability

All data from this study are presented in the paper.

## Abbreviations

SylSR	Sylgard 184 silicone rubber
HTVSR	hot vulcanized silicone rubber
STF	shear thickening fluid
MVMQ	methyl vinyl silicone rubber
DCP	dicumyl peroxide
OM	optical microscope
FE-SEM	field-emission scanning electron microscopy
DMA	dynamic thermo-mechanical analysis
